# Identification of the Chemical Forms of Heavy Metals in Municipal Sewage Sludge as a Critical Element of Ecological Risk Assessment in Terms of Its Agricultural or Natural Use

**DOI:** 10.3390/ijerph17134640

**Published:** 2020-06-28

**Authors:** Malwina Tytła

**Affiliations:** Institute of Environmental Engineering, Polish Academy of Sciences, 34 M. Skłodowskiej-Curie St., 41-819 Zabrze, Poland; malwina.tytla@ipis.zabrze.pl; Tel.: +48-32-271-6481 (ext. 135)

**Keywords:** heavy metals, sewage sludge, ecological risk assessment, risk indices, sequential extraction, chemical forms of heavy metals, environmental pollution, multivariate statistical analysis

## Abstract

The present study aimed to demonstrate that identification of the chemical forms of heavy metals in sewage sludge produced in municipal Wastewater Treatment Plants (WWTPs) is a critical element of ecological risk assessment, especially in terms of its agricultural or natural use. The concentrations of seven heavy metals (Cd, Cr, Cu, Ni, Pb, Zn and Hg) were determined using inductively coupled plasma optical spectrometry (ICP-OES) and cold vapor atomic absorption spectrometry (CV-AAS). The chemical forms of heavy metals were analyzed in accordance with the sequential extraction method proposed by the Community Bureau of Reference (BCR). Sludge samples were collected at the five municipal WWTPs located in the largest industrial area in the country, i.e., the Upper Silesian Industrial Region (southern Poland, central Europe). The ecological risk was assessed by calculating the Potential Ecological Risk Factor (ER), Risk Index (RI), Risk Assessment Code (RAC), Individual Contamination Factor (ICF), Global Risk Index (GRI) as well as the author’s indices, i.e., Individual Ecological Risk (IER) and Global Ecological Risk (GER). To demonstrate the differences between the level of ecological risk posed by the different heavy metals, sludge samples were collected at two specific points of the processing line. Considering the chemical forms of heavy metals, the highest ecological risk was posed by Zn, Cd and Ni, while in the case of their total concentrations, by Cd and Hg. The obtained results confirm that quantitative determination of the total content of heavy metals in sewage sludge is not a sufficient criterion in assessment of the ecological risk that these elements pose to the natural environment and living organisms. Moreover, multivariate statistical analysis revealed a significant correlation between the concentrations of heavy metals, which indicates that they plausibly originate from the same source of pollution.

## 1. Introduction

For many years, an increase in the amount of sewage sludge generated in municipal Wastewater Treatment Plants (WWTPs) has been observed. The main reason for this phenomenon is population growth and the increasing effectiveness of biological wastewater treatment processes used in WWTPs [1,2]. The disposal of sewage sludge is nowadays one of the most important issues in wastewater management, in the majority of European countries. The Silesian Voivodeship (southern Poland, central Europe) is one of the most urbanized and polluted regions in Poland, with an enormous concentration of industries, including hard coal mining, electric power industries, transportation, etc. The most crucial place in this area is the Upper Silesian Industrial Region (in Polish: Górnośląski Okręg Przemysłowy; GOP). This is the largest industrial area in the country and one of the biggest in Europe, a so-called key anthropogenic “hot spot” area [3,4,5]. In 2018, in the Silesian Voivodeship there were 201 municipal WWTPs in operation, among which 109 were biological and 91 with increased biogene removal. The amount of generated sewage sludge was 65,900 Mg of dry matter (DM), which puts the Silesian Voivodeship in 3rd place in the country in terms of the amount of sludge produced. According to Poland’s largest database, the “Local Data Bank”, 3036 Mg_DM_ of municipal sludge produced were used in agriculture; 2230 Mg_DM_ for land reclamation; 5535 Mg_DM_ to grow plants for compost production; 4170 Mg_DM_ were thermally treated; 251 Mg_DM_ deposited; and 1541 Mg_DM_ temporarily stored [6]. It is estimated that by 2022 the amount of sludge produced in this region will reach 74,760 Mg_DM_ [7]. 

The major methods for sludge disposal are agriculture and land reclamation (agricultural purposes), as well as cultivation of plants intended for compost production, reclamation or adaptation of lands to specific needs, cultivation of crops not intended for the consumption or production of animal feed (natural purposes), as well as incineration and landfilling [1,8,9,10]. The most popular method is the application of the sludge to the land, both for agricultural and natural purposes [9,10]. This is the most economically attractive method, which is characterized by low costs and high efficiency. For these reasons, it has been successfully used in Poland and all over the world [9,11]. Moreover, sewage sludge contains organic matter and other substances needed by plants for growth, e.g., nitrogen, phosphorus, potassium, calcium and magnesium [1,11,12,13,14,15], and thus it is successfully used in agriculture as fertilizer or a regenerator for soil [11,16]. Unfortunately, as being the final waste material of wastewater treatment, sewage sludge also contains pathogens, poorly biodegradable organic compounds as well as heavy metals (including those toxic), etc. Among the above-mentioned hazard factors, the content of heavy metals constitutes one of the greatest restrictions on use of sludge for agricultural or natural purposes [1,4,16,17]. Heavy metals in the sewage sludge originate from domestic and industrial wastewater, surface runoff, corrosion of sewerage system, pharmaceuticals, body care and cleaning products, as well as from illegal discharges, etc. [2,18,19]. In Poland, the concentration limit values of heavy metals in sewage sludge are specified in the Regulation of the Minister of Environment of 6 February 2015 (J. L. 2015, Item. 257) [8], whereas in the European Union (EU), to which Poland belongs, in the Council Directive of 12 June 1986 (86/278/EEC) [20]. However, it must be emphasized that processes commonly used in municipal WWTPs do not guarantee definite heavy metal removal [21]. So, application of sewage sludge to land solves the problem of its final disposal, but on the other hand may pose the potential risk associated with the secondary contamination of soil and ground water by heavy metals present in the sludge [12]. Moreover, introduction of toxic elements into the natural environment might negatively affect microorganisms, plants, animals and finally human health [11,22]. 

It is commonly known that knowledge of the total concentrations of heavy metals in sewage sludge allows only for an evaluation of their pollution degree, but is not conclusive, with potential ecological risk that these elements may pose to the environment and living organisms, including humans [2]. This is due to the fact that the mobility, bioavailability and toxicity of these elements depend on their speciation forms, which are influenced by their leaching and interactions with different components of the natural environment [1,2,14,22,23]. One of the most popular methods for determination of the chemical forms of heavy metals in environmental samples (sediments, soils and sewage sludge), is the three-step chemical sequential extraction procedure, proposed by the Community Bureau of Reference (BCR), now the Standards, Measurements and Testing Programme [24,25]. This procedure has arisen as a result of modification of the Tessier method [26]. BCR sequential extraction allows to determine the constituents of the sludge to which particular heavy metals are bound to, and enables a detailed assessment of the ability of these elements to migrate from the sludge to the soil and ground water, and finally to the plants [4,16]. In other words, this method provides the detailed data necessary for an ecological risk assessment. The knowledge of the chemical forms of heavy metals in sewage sludge is extremely important, due to the fact that some of the soil parameters, such as pH, potential redox, organic matter, etc., may influence their mobility and bioavailability after sludge application [15,19,23]. Therefore, to demonstrate that identification of the chemical forms of heavy metals in sewage sludge is a critical element of ecological risk assessment, two different groups of indices were compared, i.e., total content and speciation indices, the so-called Ecological Risk Assessment Methods (ERA). The first group includes the Potential Ecological Risk Factor (ER) and Risk Index (RI) [27], whereas the second one the Risk Assessment Code (RAC) [28], Individual Contamination Factor (ICF) [29,30] and Global Risk Index (GRI) [30], as well as the Individual Ecological Risk (IER) and Global Ecological Risk (GER) (the author’s indices). The above indices include various aspects. For example, both ER and RI focus on the total quantity of heavy metals and their toxicity; RAC, ICF, IER and GER focus on heavy metal mobility, while GRI includes both mobility and toxicity [2]. The most commonly used indices are ER, RI and RAC [2,9,31,32], while ICF and GRI are rarely applied [2,33]. The above-listed indices (except for IER and GER) were also used to assess the ecological risk in other environmental samples, like soils and sediments [5,30,34]. So far, there have been only a few studies regarding the identification of the chemical forms of heavy metals in sewage sludge at different stages of its processing [2,4,35]. Unfortunately, the overwhelming majority of current studies concerns only the dewatered sewage sludge [14,16,17,31]. Therefore, in the present research, two types of sewage sludge were examined, i.e., thickened and dewatered and/or hygienized, collected at the beginning and final stage of the processing line, respectively. To the author’s knowledge, this is the first such a detailed study that concerns assessment of the ecological risk of heavy metals in sewage sludge from several WWTPs located in the Upper Silesian Industrial Region (Poland), which is one of the biggest key anthropogenic “hot spot” areas in Europe. 

The main objectives of this study are (1) to evaluate the concentrations of Cd, Cr, Cu, Ni, Pb, Zn and Hg in sewage sludge samples from five municipal WWTPs (Upper Silesian Industrial Region; southern Poland) in terms of the current regulations on the application of sludge into the land; (2) to investigate the chemical forms of heavy metals in sludge samples by using the BCR sequential extraction procedure for assessing its mobility and bioavailability, including sludge characteristics at the initial and final stage of its processing; (3) to assess the ecological risk of heavy metals in sewage sludge; (4) to demonstrate that identification of the chemical forms of heavy metals in sewage sludge is a critical element of risk assessment; and (5) to find relationships between the heavy metals and ecological risk levels, as well as to identify their possible sources (by using multivariate statistical analysis).

## 2. Materials and Methods 

### 2.1. Study Area

**Figure 1:**
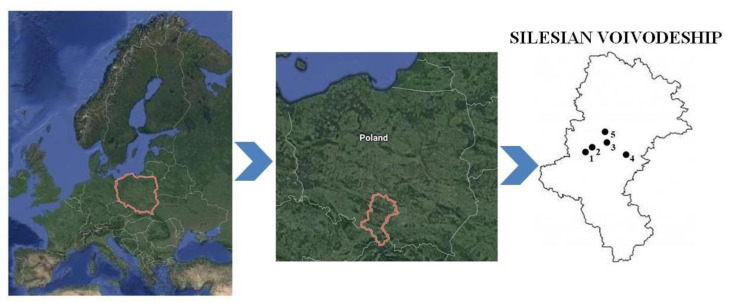
Map of the study area and location of the Wastewater Treatment Plants (WWTPs).

The study area, the Upper Silesian Industrial Region (in Polish: Górnośląski Okręg Przemysłowy; GOP) is mainly located in the central-eastern part of the Silesian Voivodeship, southern Poland. This is the largest industrial area in the country, and one of the biggest in Europe. The most dominant industrial sectors in the Upper Silesian Industrial Region are mining, iron and steel, transport, energy, coke engineering, mechanical engineering, chemical industry and building materials [3,5]. The variety of industrial activities in the study area contributes to high environmental pollution, especially with heavy metals, which has a crucial impact on the composition of raw wastewater flowing into the municipal WWTPs, and finally on the physicochemical characteristics of the sewage sludge produced [2,4]. A map of the study area and the locations of the WWTPs are shown in Figure 1.

### 2.2. Sewage Sludge Sampling

Sewage sludge samples used in this study were collected at the five municipal WWTPs (WWTP1-WWTP5), located in different sites of the Upper Silesia Industrial Region (Silesian Voivodeship, southern Poland). Sludge samples were collected at the two stages of its processing line, i.e., at the beginning—before digestion (the mixture of primary and secondary sludge after thickening; S1) and at the end (digested sludge after dewatering and/or hygienization; S2). In two of the five considered WWTPs, the dewatered sewage sludge was not subjected to the process of hygienization (WWTP4 and WWTP5). All samples were collected twice, at 1-h intervals. This was intended to obtain the most representative samples for research. Digested sludge was collected after a period of time necessary for its stabilization in a given WWTP (about 20–32 days). All the WWTPs have a similar technological line and conduct mechanical and biological processes of wastewater treatment, as well as anaerobic digestion of sewage sludge. Collected samples were kept in polypropylene containers and stored in a refrigerator at 4 °C until laboratory analysis. The operational parameters of the investigated WWTPs are shown in Table 1.

### 2.3. Methods for Physicochemical Analysis of Sewage Sludge 

#### 2.3.1. Sewage Sludge Analysis

The dry (DM) and organic matter (OM) were analyzed according to Polish Standards (PN-EN 12880:2004 and PN-EN 12879:2004) [36,37]. The pH and potential redox (Eh) of the sewage sludge were measured using a multifunctional meter, CPR-411 (Elmetron), equipped with two electrodes, IJ44A and ERS-2 (Elmetron), respectively. Sample analysis involved determination of the total concentrations and chemical forms of the selected heavy metals (Cd, Cr, Cu, Ni, Pb, Zn and Hg) in the sewage sludge, collected at two stages of its processing line (S1 and S2). The total heavy metal concentrations in the obtained samples and extracts were determined using inductively coupled plasma optical spectrometry (Avio 200 ICP-OES, PerkinElmer Inc., Waltham, MA, USA). Mercury was assayed with cold vapor atomic absorption spectrometry (CV-AAS). The limits of quantifications (LOQs) were 0.015, 0.019, 0.025, 0.020, 0.027, 0.024 and 0.0005 mg·L^−1^ for Cd, Cr, Cu, Ni, Pb, Zn and Hg, respectively. All measurements were performed in triplicate.

#### 2.3.2. Sample Preparation for the Determination of Heavy Metals in Sewage Sludge

The sample preparation for the total determination of heavy metals (Cd, Cr, Cu, Ni, Pb, Zn and Hg) in two types of sewage sludge included the following steps: (1) initial drying of sewage sludge for 48 h at room temperature until air-dried and then to a constant mass at 105 °C; (2) grinding of dry sludge in a mortar grinder; and (3) digestion of 0.2 g of sludge sample with 15 mL of 35% HCl and 5 mL of 65% HNO_3_ (aqua regia). The mixture was placed in glass flask (class A) and heated on an electric hot plate. After cooling, the obtained solutions were filtered through fine filters (0.45 µm) and diluted with 5% HNO_3_ to a volume of 50 mL. All the sludge samples were stored at 4 °C until laboratory analysis. Standards were prepared on the day of analysis. 

#### 2.3.3. Sequential Extraction of Heavy Metals in Sewage Sludge

The BCR three-step sequential extraction procedure proposed by the Community Bureau of Reference (now the Standards, Measurements and Testing Programme) was used for the identification of the chemical forms of the heavy metals in the sludge samples (F1–F3). After the end of the sequential extraction steps, the residual heavy metal contents were determined (additional step; F4). The recovery rate (R; %) in the sequential extraction procedure was calculated by comparing the sum of the four fractions with the total concentrations of the elements in the sludge samples, after digestion with aqua regia. A scheme of the BCR sequential extraction procedure for the fractionation of heavy metals in sewage sludge is shown in Table 2. 

### 2.4. Quality Control

In order to check the accuracy and precision of the method used for total heavy metals determination in sewage sludge, the certified reference material ERM-CC144 (JRC) was used. Sample digestion was carried out in triplicate. The recovery rates for the heavy metals in the reference material were between 83% and 103%, which indicate that the conducted analysis was under control. The average values of the relative standard deviation (RSD; %) were less than 10% for each of the considered heavy metals, which is satisfactory for environmental analysis. The results of the heavy metal concentrations in the certified reference material are shown in Table 3.

### 2.5. Ecological Risk Assessment 

The two total content indices (ER and RI) and five speciation indices (RAC, ICF, IER, GRI and GER) were used to assess the ecological risk of seven heavy metals in two types of sewage sludge from five municipal WWTPs. Moreover, two of the above-listed indices were proposed by the author, namely, IER and GER. 

Development of the author’s indices was possible thanks to the data obtained within this study, as well as previous observations [2,4]. Moreover, according to data in the literature, heavy metals associated with the fractions F1 (acid soluble/exchangeable) and F2 (reducible) are considered to be most mobile in the environment. This is due to the fact that the heavy metals that bound to these fractions are easily dissolved in soil solutions and assimilated by plants [2,4,16,35]. However, the calculation method of RAC, which is one of the most popular indices used for ecological risk assessment, does not include the content of heavy metals in fraction F2, while the results from the present study indicate that selected elements (Zn, Cd and Ni) are strongly bound to the reducible fraction (F2). Taking into account the above-mentioned facts, it was stated that it is necessary to develop new indices, which will include the concentrations of heavy metals in both the mobile fractions. Therefore, two indices were proposed, namely IER and GER. The first one refers to a single heavy metal, whereas the second one to the group of heavy metals.

The indices were calculated using the equations presented in Table 4.

### 2.6. Statistical Analysis

All statistical analyses were performed using Statistica 13 Package (StatSoft, Poland). Pearson’s correlation coefficients (r) were calculated to determine the relationships between the different heavy metals and physicochemical parameters of the sludge samples. A similar analysis was also performed in relation to the levels of risk, calculated for different heavy metals in the sludge samples. Cluster Analysis (CA) was applied to group the heavy metals from the different WWTPs into meaningful groups (clusters), and the Ward method was used for data agglomeration. Factor Analysis (FA) was applied to obtain more reliable information about the relationships between the different heavy metals in the sludge samples. To extract the significant components and identify the possible sources of heavy metals, a Principle Component Analysis (PCA) along with a Varimax rotation (with Kaiser normalization) was carried out. All statistical analyses were performed at a 95% confidence interval (*p* < 0.05). Data analysis also included calculating the mean ($\bar{x})$ and standard deviation (SD).

## 3. Results and Discussion

### 3.1. Total Concentrations of Heavy Metals in Sewage Sludge

The physicochemical characteristics of the sewage sludge from the five municipal Wastewater Treatment Plants (WWTP1–WWTP5) are summarized in Table 5. It was found that the sludge samples collected at the initial stage of its processing (S1) are characterized by lower pH values (6.0–6.8), compared to those from the final stage (S2) (8.1–8.9). The higher pH values in the samples collected at the final stage of sludge processing probably resulted from two causes. The first one is the need for maintaining the value of the sludge pH during the anaerobic digestion above 6.5, in order to avoid a process breakdown. The second cause is the addition of chemical agent for sludge hygienization [38]. The increase in the value of the sludge pH is a positive phenomenon in the context of its agricultural or natural use. Similar observations have been made regarding the potential redox (Eh), whose values were in the range of −114.0 to −336.0 mV and −235.5 to −349.0 mV, for sludge samples collected at the sampling points S1 and S2, respectively. In the case of dry and organic matter, an increase in their concentrations was also observed. The dry matter content ranged from 31.7 to 55.5 g·kg^−1^ and 166.4 to 269.9 g·kg^−1^ for thickened (S1) and dewatered and/or hygienized (S2) sewage sludge, respectively, whereas the organic matter content ranged from 24.0 to 41.2 g·kg^−1^ (S1) and 102.6 to 148.4 g·kg^−1^ (S2). The obtained results are consistent with those presented in other scientific studies [2,4,11,35]. However, it should be mentioned that the content of organic matter decreases during anaerobic digestion of the sludge, and then, as a result of the subsequent processes of its treatment, i.e., dewatering and/or hygienization, increases again. The above statement is in good agreement with the author's previous observations [2,4]. The probable reason for the increase in the organic matter content is the addition of a conditioning agent (polymer) during sludge dehydration [38].

According to the literature data, the concentrations of particular heavy metals in sewage sludge can be ordered as follows: Zn > Cu > Cr > Ni > Pb > Cd (the order does not include Hg) [39]. The conducted research showed that the mean concentrations of the heavy metals in the sewage sludge collected at the initial stage of its processing (S1) were in the following order: Zn > Cu > Ni > Cr > Pb> Cd > Hg (WWTP1); Zn > Cu > Pb > Cr > Ni > Cd > Hg (WWTP2); Zn > Cu > Pb > Ni > Cr > Cd > Hg (WWTP3); Zn > Cu > Cr> Pb > Ni > Cd > Hg (WWTP4); and Zn > Pb > Cu> Cr > Ni> Cd > Hg (WWTP5). A similar tendency was observed in relation to sewage sludge taken from the final stage of its treatment (S2). The only exception was sludge samples from WWTP3 and WWTP4, where a light difference in Pb, Ni and Cr concentrations were observed, i.e., Zn > Cu > Pb > Cr > Ni > Cd > Hg (WWTP3) and Zn > Cu > Pb > Cr > Ni > Cd > Hg (WWTP4). Comparing the heavy metal concentrations in samples collected at the initial and final stage of sludge processing in the examined WWTPs, no significant effect of the hygienization process was observed. The most dominant heavy metals in both types of analyzed sludge were Zn and Cu, whereas Cd and Hg were in the lowest concentrations. The only exception was sewage sludge collected at the final stage of its processing in the WWTP5, where Zn and Pb were in the highest concentrations. The obtained results are in good agreement with those presented in the scientific literature [2,4,9,23]. For example, the concentrations of heavy metals in thickened and dewatered sludge samples from the selected municipal WWTPs in Silesian Voivodeship (southern Poland) ranked in the following order: Zn > Cu > Pb > Cr > Ni > Cd > Hg [4]. In contrast to the results presented above, other researchers reported that the content of heavy metals in sewage sludge from a municipal WWTP in Beijing (China) were ordered as follows: Zn > Cr > Ni > Cu > Pb, for thickened and dewatered sewage sludge, respectively [35]. This is probably due to the different share of industrial wastewater in the raw wastewater stream flowing into the above-mentioned WWTPs, which affects the metals content in the produced sewage sludge.

The content of heavy metals in sewage sludge depends on several factors: (1) the concentrations of these elements in wastewater discharged into the WWTP; (2) the technology for wastewater treatment; and (3) the methods of sludge processing [19]. Taking into account the high environmental pollution and diversity of industrial sectors in the study area, the content of these elements in the sewage sludge probably resulted from the difference in wastewater characteristics entering each WWTP. The above hypothesis was confirmed by other scientists, who showed that dewatered sludge from WWTPs located in the non-industrial catchment area (Warmia and Mazury, northern Poland) were characterized by a lower content of heavy metals, compared to those presented in this study, namely, Cd (1.1–1.9 mg·kg^−1^); Cu (132.4–242.6 mg·kg^−1^); Ni (9.8–91.5 mg·kg^−1^); Pb (8.0–17.9 mg·kg^−1^) and Zn (564.2–994.5 mg·kg^−1^) [14]. Moreover, the conducted research showed that according to Polish regulations, the content of Zn in the sewage sludge (S2) from WWTP4 exceeded the permissible concentration value for this element—in case of use of sludge in agriculture [8]. It means that it cannot be directly applied to agricultural land, but it can still be used for natural purposes, e.g., for reclamation or adaptation of lands to specific needs. In contrast to Polish regulations, according to the European Union standards, the content of Zn in the discussed sewage sludge meets the requirements of the Council Directive of 12 June 1986 (86/278/EEC) [20], by which the permissible concentration value of this heavy metal in sludge used in agriculture is in the range of 2500–4000 mg·kg^−1^. Therefore, the decision on authorization of the above-mentioned sludge for use in agriculture should to be taken by the relevant authorities.

### 3.2. Chemical Forms of Heavy Metals in Sewage Sludge

**Figure 2:**
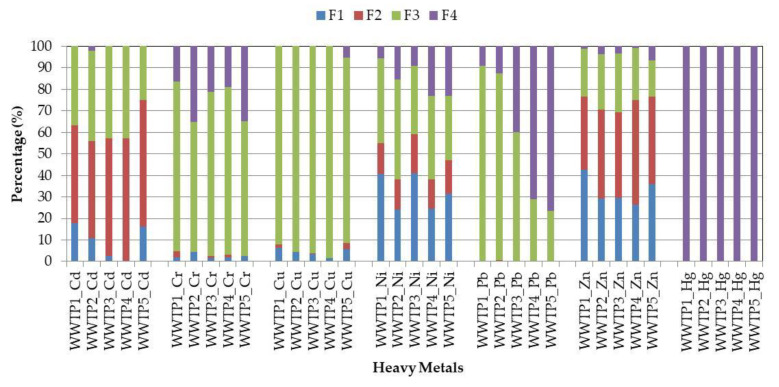
The average percentage distribution of each heavy metal fraction in the thickened sludge samples (S1).

**Figure 3:**
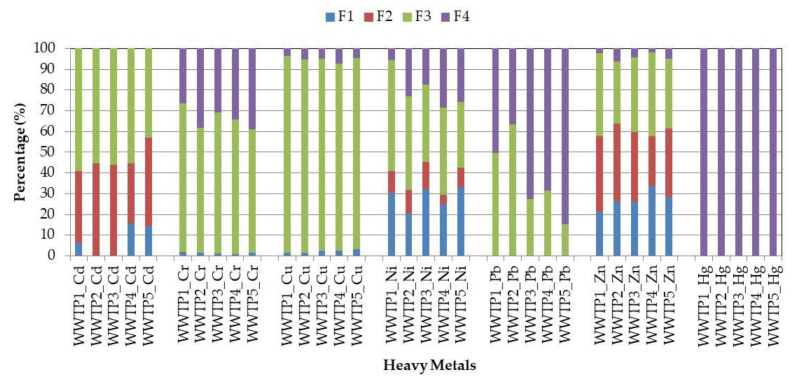
The average percentage distribution of each heavy metal fraction in the dewatered and/or hygienized sewage sludge samples (S2).

The concentrations of the heavy metals in the individual fractions of the sewage sludge from the five municipal WWTPs are shown in Table 6, whereas their chemical speciation is represented as the percentage of total content is shown in Figure 2 and Figure 3. Verification of the BCR sequential extraction procedure were conducted with using the recovery rate (R; %). With respect to the Cd, Cr, Cu, Ni, Pb and Zn content, the recovery rate was in the range of 71.1–129.5% and 83.1–146.1% for the sludge samples collected at the sampling points S1 and S2, respectively. The above results are in good agreement with those presented in the author’s previous research, where the recovery rate for the same heavy metals amounted to 52.9–114.1% (thickened sludge) and 41.4–119.2% (dewatered sludge) [2]. Furthermore, a similar result in relation to dewatered sludge was also reported by other researchers, i.e., 90.3–130.9% [14], 78.4–106.1% [23] and 72.0–123.9% [31]. The above findings confirm that the BCR sequential extraction used for detecting the chemical speciation of Cd, Cr, Cu, Ni, Pb and Zn in the examined sewage sludge was exact and reliable. However, the conducted research also revealed that the recovery rate of mercury for the thickened sludge samples was lower (34.8–56.7%) than this for the dewatered and/or hygienized ones (31.2–113.1%). The above findings indicate that BCR sequential extraction may not be reliable for determination of the chemical speciation of mercury in every type of sewage sludge since each extraction step influences its volatilization and removal from the samples. Strongly reducible conditions are needed for Hg capturing. However, further research should be conducted to clarify this issue. Generally, it was found that sewage sludge collected at different stages of its processing exhibited higher concentrations of selected heavy metals bound to the immobile fractions (F3 and F4). The only exception was zinc, as well as cadmium and nickel (in selected WWTPs), in which case the percentage share in the mobile fractions (F1 + F2) were higher than those in the immobile ones. The obtained results are also confirmed by other researchers, who indicate that Zn, Cd and Ni in dewatered sludge were bound to the mobile fractions, i.e., 79.1% (Zn), 51.4% (Cd) and 70.3% (Ni) [31]. Similar observations were partly made in relation to thickened sludge, for which the percentage share of Zn in the mobile fractions reached 62.5% [35]. The high percentage share of zinc, cadmium and nickel in fractions F1 and F2 is probably associated with their fractional composition in wastewater flowing into the WWTPs, or results from the changes in the characteristics of sewage sludge during its treatment. However, further research should be conducted to explain this issue. Moreover, the conducted research also revealed that the concentrations of the mobile heavy metals in the thickened sludge samples (S1) were higher than those in the dewatered and/or hygienized ones (S2). The percentage share of Zn, Cd and Ni in the samples collected from the initial and final stage of sludge processing was as follows: 69.2–76.7% (Zn); 57.1–74.9% (Cd); 38.0–59.2% (Ni); 57.5–63.7% (Zn); 40.8–57.1% (Cd); 29.4–45.4% (Ni); while for the other elements very low (Figure 2 and Figure 3). Similar results also have been reported in the author’s previous research [2,4]. For all WWTPs, chromium and copper, in both types of sewage sludge, were distributed in fraction F3; cadmium in fractions F3 and F4; while mercury only in fraction F4. The above conclusions are in good agreement with the results obtained by other researchers [9,11,31]. Moreover, the distribution of particular heavy metals bound to the mobile fractions was as follows: Zn > Cd > Ni > Cu > Cr > Pb = Hg (WWTP1 and WWTP5); Zn > Cd > Ni > Cr > Cu > Pb = Hg (WWTP2 and WWTP4); Zn > Ni > Cd > Cu > Cr > Pb = Hg (WWTP3) and Zn > Cd > Ni > Cr > Cu > Pb = Hg (WWTP1 and WWTP2); Zn > Ni > Cd > Cd > Cu > Cr > Pb = Hg (WWTP3); and Zn > Cd > Ni > Cu > Cr > Pb = Hg (WWTP4 and WWTP5), for sewage sludge collected at the sampling points S1 and S2, respectively. The obtained results confirm that the processes used for sludge treatment have a crucial impact on the distribution of heavy metals in the chemical fractions. The percentage share of the analyzed heavy metals in the mobile fractions of the sludge samples collected at the final stage of its processing was lower than in the thickened ones. This means that processes of anaerobic digestion, dehydration and hygienization have a positive impact on reducing the mobility of the heavy metals in the sewage sludge. The above observations are particularly important in the case of agricultural or natural use of municipal sewage sludge. In conclusion, the conducted research confirms that the total heavy metal content in sewage sludge should not be the most crucial guideline when choosing the way of its disposal. Moreover, the main criterion should be knowledge of their chemical forms.

### 3.3. Ecological Risk Assessment of Heavy Metals in Sewage Sludge

The first group of ecological risk indices used in this study refers to the total content of heavy metals in the analyzed sewage sludge samples. The results of the ecological risk assessment according to the total content indices are shown in Table 7 and Table 8. Taking into account that in the present study two types of sewage sludge were analyzed, it was assumed that the thickened sludge describes an “initial risk”, whereas dewatered and/or hygienized ones the “final risk”. The mean values of the Potential Ecological Risk Factor (ER), calculated for seven heavy metals in the thickened sludge samples (S1) from five municipal WWTPs, were in the decreasing order of Cd > Hg > Ni > Zn > Pb = Cu > Cr (WWTP1); Cd > Hg > Pb > Zn > Cu > Ni > Cr (WWTP2, WWTP3 and WWTP5); and Cd > Hg > Zn > Pb > Cu > Ni > Cr (WWTP4). Similar observations were made in relation to sludge samples taken from the final stage of its processing (S2). The above findings indicate that cadmium and mercury posed the highest ecological risk to the natural environment and living organisms, including humans. The ER values for these elements were in the range of 435.0–1384.8 (Cd), 103.0–231.5 (Hg), 557.8–2082.9 (Cd) and 124.4–691.1 (Hg) for the thickened and dewatered and/or hygienized sludge samples, respectively. Similar observations were made by other scientists, who indicated that the values of the ER calculated for the selected heavy metals in the dewatered sludge samples collected at the three WWTPs in China, were ranked in the following order: Cd > Hg > Cu > As > Ni > Pb > Zn > Cr [9]. Furthermore, other scientific papers also confirm that cadmium poses the highest ecological risk; for example, ER: 4150.9–6521.6 [31] and ER: 125.0–344.6 [32]. As can be noted in the present study, higher values of ER were indicated for sludge samples taken from the final stage of its processing. This means that the “initial risk” was lower that the “final risk”. This was related to the higher concentrations of heavy metals in the dewatered and/or hygienized sludge samples. The obtained results are in good agreement with the author’s previous research [2]. Moreover, to quantify the total ecological risk of the heavy metals in both types of sewage sludge, the Risk Index (RI) was calculated. The values of RI ranged from 576.4 to 1581.8 and 763.1 to 2333.8, for sludge samples collected at the sampling points S1 and S2, respectively. The obtained results revealed that regardless of the type of sewage sludge, the considered heavy metals posed a high ecological risk. The mean values of RI for sewage sludge originated from the five municipal WWTPs formed the following series: WWTP5 > WWTP3 > WWTP4 > WWTP1 > WWTP2 and WWTP5 > WWTP4 > WWTP3 > WWTP1 > WWTP2, for sludge samples collected at the initial and final stage of its processing, respectively. The above observations are in good agreement with the results presented by other researchers, who indicated that the RI values of heavy metals in dewatered sludge were in the range of 1254.0–1469.1 [9]. Moreover, it is important to note that ER and RI relate to the total content of heavy metals in the sewage sludge, as well as to their toxicity. Unfortunately, it is still not enough to predict what will happen after application of the sewage sludge to land. For this reason, it is necessary to identify the chemical forms of the heavy metals, which will allows determining their mobility and bioavailability in sewage sludge before its agricultural or natural disposal. 

The second group of risk indices used in this research refers to the chemical forms of the heavy metals in sewage sludge. Each index is calculated in a different way, which makes it possible to conduct a comprehensive and highly credible risk analysis. The results of the ecological risk assessment according to the speciation indices are shown in Table 7 and Table 8. According to the RAC guidelines, it can be noted that the percentage shares of heavy metals associated with the fraction F1 in the examined sludge samples varied in the order of Zn > Ni > Cd > Cu > Cr > Pb = Hg (WWTP1 and WWTP5); Zn > Ni > Cd > Cr > Cu > Pb = Hg (WWTP2); Ni > Zn > Cu > Cd > Cr > Pb = Hg (WWTP3); Zn > Ni > Cr > Cu > Cd = Pb = Hg (WWTP4) and Ni > Zn > Cd > Cu > Cr > Pb = Hg (WWTP1 and WWTP5); Zn > Ni > Cd > Cr > Cu > Pb = Hg (WWTP2); Ni > Zn > Cu > Cr > Cd > Pb = Hg (WWTP3); and Zn > Ni > Cd > Cu > Cr > Pb = Hg (WWTP4), for sludge collected at the sampling points S1 and S2, respectively. Among them, Zn, Ni and Cd posed the highest potential risk to the plants and living organisms. The percentage shares of these elements in mobile fractions were as follows: 26.2–42.5% (Zn), 24.1–41.1% (Ni), 2.4–17.7% (Cd), 21.4–33.9% (Zn), 20.3–33.2% (Ni) and 6.2–16.4% (Cd), for sludge samples collected at the initial and final stage of its processing, respectively. These findings are in good agreement with the results obtained by other researchers, who found that the most environmentally hazardous heavy metals in sewage sludge were zinc (RAC: 39.0–52.9%), nickel (RAC: 37.1–43.0%) and cadmium (RAC: 17.7–23.2%) [9].

The mean values of another speciation index, i.e., the Individual Pollution Index (ICF), indicate that regardless of the sludge sampling point, heavy metals pose different levels of ecological risk. Taking into consideration the obtained results, it was revealed that ICF may not be an entirely authoritative tool for analysis of the ecological risk of heavy metals in sewage sludge. This is due to its calculation formula. The ICF value is calculated as a quotient of the content of a given heavy metal in fractions F1–F3 and F4. According to the literature data, fraction F3 is considered an immobile one, so this method can give not entirely reliable results [16,35]. In addition, it is also impossible to calculate the level of ecological risk when a given element does not occur in the residual fraction (F4). This was particularly evident in relation to Cd. Therefore, it was decided that the results of the risk analysis performed by using the ICF index will not be considered if the given heavy metal is bound mainly in the third fraction or if it is not found in the residual one. Taking into account the above assumptions, only Zn (ICF: 14.5–121.7) and Ni (ICF: 3.3–16.8) were potentially environmentally hazardous. Moreover, to quantify the total ecological risk of the chemical forms of heavy metals, the GRI was used. Unfortunately, the GRI is based on the ICF value, so this index does not seem to be an entirely representative tool for risk analysis as well. For this reason, the GRI was used only to assess the ecological risk of Zn and Ni, whose levels ranged from 31.2 (LR) to 181.5 (MR) and 31.9 (LR) to 124.8 (LR) for sewage sludge collected at the sampling points S1 and S2, respectively. The above results indicate that generally these two heavy metals posed from a low to a moderate ecological risk. In contrast to the above findings, other scientists indicated that the sewage sludge collected from WWTP in Urumqi (Xinjiang Province, China) were slightly polluted by Cd, Cr, Cu, Ni, Pb and Zn (GRI: 132.9), which means that these elements did not pose a serious threat to the environment and living organisms [33]. However, it should be emphasized that, so far, both ICF and GRI have rarely been used in relation to sewage sludge.

According to the author’s method, the mean values of the Individual Ecological Risk (IER) for each of the examined heavy metals indicated that zinc, cadmium and nickel posed the highest ecological risk, i.e., Zn (IER: 225.2–329.4%), Cd (IER: 127.0–298.4%), Ni (IER: 61.4–145.2%), Zn (135.6–175.2%), Cd (IER: 68.9–133.2%) and Ni (IER: 69.1–83.1%) for thickened and dewatered and/or hygienized sludge samples, respectively. Other elements did not pose a threat to the natural environment, plants, animals or humans. The above-presented results are partly consistent with those obtained for RAC. Moreover, considering the mean values of another one of the author’s indices, the Global Ecological Risk (GER), it was confirmed that globally the heavy metals examined in this study pose a high environmental risk. The GER values were in the range of 438.1–723.7% and 261.8–372.7%, in relation to sewage sludge collected at the sampling points S1 and S2, respectively.

In summary, Zn, Cd and Ni were selected as the most hazardous heavy metals, due to their high mobility and bioavailability, in both types of examined sewage sludge. The mean values of the speciation indices calculated to assess the level of ecological risk of the heavy metals in the analyzed sludge samples presented a wide range in all municipal WWTPs. The obtained results indicate that considering the chemical forms of the heavy metals, the higher ecological risk is posed by the thickened sewage sludge (S1). This means that, in general, the level of risk associated with the presence of heavy metals in thickened sludge (“initial risk”) decreases in the subsequent stages of its processing, which is a positive phenomenon in the context of agricultural or natural use of sewage sludge. This is probably associated with the changes in sludge characteristics (mainly pH value), resulting from the various processes carried out in the WWTPs (in particular the digestion process). The conducted research also revealed that some of the ERA methods (ICF and GRI) may not be entirely authoritative tools for analysis of the ecological risk of heavy metals in sewage sludge. Further research on this subject should be carried out. Moreover, considering the indices used in the present study, it can be assumed that RAC, IER, ER, RI and GER are the most reliable tools for assessment of the ecological risk posed by heavy metals present in different types of sewage sludge.

### 3.4. Multivariate Statistical Analysis

The multivariate analysis was carried out to find the relationships between the different variables. The traditional statistical approaches, such as Pearson’s correlation, Factor Analysis (FA), Principal Component Analysis (PCA) and Cluster Analysis (CA), were the effective tools used in determining the relationships between the different variables, as well for identification of the pollution sources [5,9,14,23,31]. 

#### 3.4.1. Relationships between Different Variables

Pearson’s correlation coefficients (r) were calculated to determine the relationships between the different variables (Table 9). The results obtained for the thickened sludge samples indicate the presence of strong correlations between Cd and Pb; Cr and Cu; Cr and Zn; Cr and Hg; Cu and Zn; and Ni and Hg. (r > 0.6). Moreover, it was also found that Cr and Cu correlate with Eh, DM and OM, while Hg with DM and OM. Similar relationships have been seen in relation to dewatered and/or hygienized sludge samples. The only difference was the strong correlation between Cu and Hg, instead of Ni and Hg. Moreover, significant correlations between Cd, Pb, Zn and DM and OM have also been noted. The obtained results are in good agreement with the experimental data presented by other researchers [9,40]. Minor differences in correlations between individual variables are probably related to the processes to which the sewage sludge was subjected. However, strong relationships between heavy metals in both types of sewage sludge may prove that they possibly originate from the same sources of pollution. In order to verify the above assumptions, further statistical analysis was carried out.

Moreover, correlation matrices were also used to determine the relationships between the levels of risk calculated for different heavy metals in the examined sludge samples (Table 10). The above analysis did not include lead and mercury, for which the levels of risk equaled zero. The obtained results indicate the presence of strong correlations between the values of RAC and IER computed with regard to the content of Cd, Cr, Cu, Ni and Cr (S1), as well as Ni, Zn and Cd (S2) in the mobile fractions of the sewage sludge samples. For the Zn and Cd in the thickened sludge samples, the correlation was strong but not significant. The above observations probably result from the addition of a conditioning agent (coagulant) to improve the effects of the sludge thickening. Another cause of weak correlation between the examined elements can be a less homogeneous structure of the thickened sewage sludge, which may affect the extraction process and in consequence the obtained results as well. However, taking into account the above results, it can be stated that, in general, the above-mentioned indices are compatible with each other, which confirms that IER is a reliable tool for ecological risk assessment. However, the performed analysis also revealed the existence of significant correlations between other indices, i.e., RAC and ER, as well as IER and ER, but they were not as strong as those between RAC and IER. Moreover, the obtained results showed that there were no significant correlations between the values of the global indices, i.e., RI, GRI and GER. The lack of a strong relationship between the GER and GRI is probably connected with the assumption that the GRI may not be an entirely authoritative tool in the analysis of ecological risk. While, in the case of the GER and RI, the correlation between these elements exists, but it is not statistically significant.

#### 3.4.2. The Source of Heavy Metals Pollution

**Figure 4:**
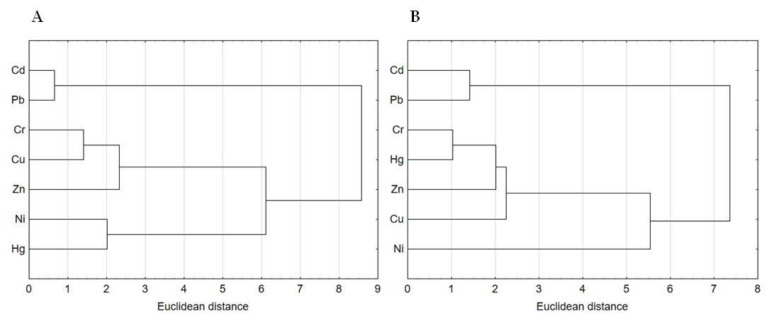
Cluster Analysis (CA) by Ward for thickened (**A**) and dewatered and/or hygienized (**B**) sewage sludge samples.

Identification of the potential sources of heavy metal pollution in municipal sewage sludge is a very important aspect of their monitoring and quality control. Figure 4A,B presents the results of the Cluster Analysis (CA), which was performed to group the heavy metals from the five WWTPs into meaningful groups (clusters). Heavy metals belonging to the same group have strong correlations among themselves and may originate from the same source of pollution. All data were standardized prior to analysis. The dendrogram for the thickened sludge samples was divided into three main groups. The first group contained Cd and Pb; the second one Cr, Cu and Zn; whereas third one Ni and Hg (Figure 4A). Moreover, the dendrogram for the dewatered and/or hygienized sewage sludge samples was also divided into three groups. The first group included Cd and Pb; the second one Cr, Hg, Zn and Cu; while third one Ni (Figure 4B). The above findings are in good agreement with the results of the Pearson correlations. Other scientists also used CA to group heavy metals into meaningful clusters [14,31].

**Figure 5:**
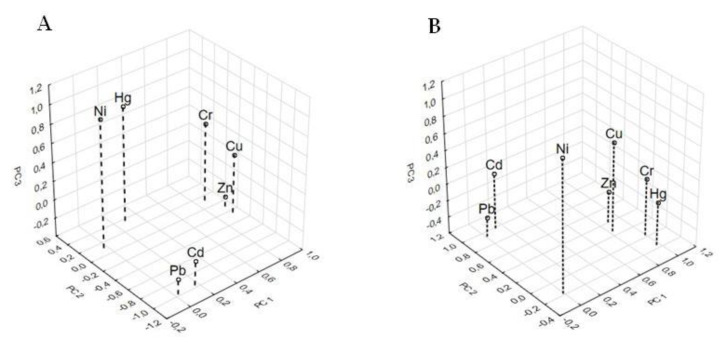
Results of the PCA corresponding to heavy metal content in thickened (**A**) and dewatered and/or hygienized (**B**) sewage sludge samples.

To obtain more reliable information about the relationships between the heavy metals in different sewage sludge samples, as well as to confirm the results obtained by the Pearson correlations and CA, a Factor Analysis (FA) was applied. To extract the significant components and identify possible sources of heavy metals in the sewage sludge samples, a Principle Component Analysis (PCA) along with a Varimax rotation was carried out. This method was used also by other researchers [23,31]. The obtained results showed that, for the thickened sludge samples collected at the five WWTPs, the Eigen values of the three principal components (PCs) were greater than 1.0 and generally accounted for 97.16% of the total variance. The variance indicates the amount of total information presented by each heavy metal. The PC1 accounted for 43.06% of the total variance and was characterized by large fractions of Cr, Cu and Zn. The second principal component (PC2) accounted for 39.0% of the total variance and included large fractions of Cd and Pb, whereas PC3 explained 15.11% and was highly loaded by Ni and Hg (Table 11). The negative loading of Cd and Pb in PC2 suggested an antagonistic effect with respect to Cr, Ni and Hg (in this component). Moreover, in the case of dewatered and/or hygienized sludge samples, similar relationships were observed. The first three PCs explained approximately 98.64% of total original data variance. The first principal component (PC1) accounted for 51.74%% of the total variance and was characterized by large fractions of Cr, Cu, Zn and Hg. The second principal component (PC2) explained 30.64% of the total variance and was highly loaded by Pb and Cd, while the third one (PC3) 16.26% and included large fraction of Ni (Table 12). The results of the statistical analysis are similar for both types of examined sewage sludges. The graphical interpretation of the PCA analysis is shown in Figure 5A,B. 

It must be emphasized that determination of the sources of heavy metal pollution should be carried out based on the content of these elements in sewage sludge collected at the initial stage of its processing (S1). This is due to the fact that the thickened sewage sludge in the most reliable way reflects the amount of heavy metals that enters the WWTPs along with the wastewater and surface runoff. The strong relationships between Cr, Cu, Zn, Cd and Pb in the thickened sludge samples, as well as the small Euclidean distances and large fractions of these elements in PC1 and PC2, indicate that they may originate from the same pollution sources. Taking into consideration the level of urbanization and industrialization of the study area, it is very difficult to identify one specific source of heavy metals. However, the most likely sources are domestic (Cr, Co, Fe, Mn and Zn) and/or industrial wastewaters (Cd, Cr, Cu, Ni, Pb, Zn and Hg); corrosion of sewerage systems (Cd and Zn) or surface runoff from urbanized areas and roads (resulting from rainfall and snowfall), which are associated with the emission of heavy metals during the transportation of vehicles (Cd, Cr, Cu, Ni, Pb, Zn and Hg); mining and smelting operations (Cd, Cu, Zn, Pb and Cr); as well as coal burning (Cd, Fe, Mn, Ni, Hg), etc. [2,18,19,41]. However, taking into account the characteristics of the studied area, it is very likely that the industry activity is the main source of wastewater pollution with heavy metals and, as a consequence, also their accumulation in sewage sludge produced in the municipal WWTPs.

## 4. Conclusions

The results of the present study showed that the concentrations of the selected heavy metals (Cd, Cr, Cu, Ni, Pb, Zn and Hg) in different types of sewage sludge—originating from five municipal WWTPs (Upper Silesian Industrial Region, southern Poland)—did not exceed the permissible levels in terms of agricultural or natural use of the sludge. This is positive information, due to the fact that three of the five examined WWTPs receive industrial effluents. Furthermore, the results of the multivariate statistical analysis indicate that the presence of heavy metals in the examined sewage sludge samples is probably because of industry activity. Considering the fractional composition of the above-listed heavy metals, it can be concluded that some of them still can be hazardous to the environment and living organisms, including humans, especially in the case of application of sludge to land. The above conclusions result from the analysis of the ecological risk, which was carried out using total content (ER and RI) and speciation indices (RAC, ICF and GERI), including those proposed by the author (IER and GER). The statistical analysis confirmed that the author’s methods are valuable. It was also revealed that some of the existing indices may not be entirely authoritative tools in the analysis of ecological risk (ICF and GRI). However, the ecological risk analysis indicated that according to the total indices, the highest risk was posed by Cd and Hg (due to its toxicity), whereas in reference to the speciation indices, the most environmentally hazardous were Zn, Ni and Cd. Taking into account that zinc, cadmium and nickel are strongly bound to the mobile fractions (F1 + F2), it is not recommended that the examined sewage sludges be applied to light and acidic soils. This is strictly related to the parameters of the above-mentioned soils, which may increase the heavy metal mobility in sewage sludge, and as a result lead to secondary environmental pollution with these elements as well. Moreover, the conducted research also revealed that the level of ecological risk referred to the chemical forms of the heavy metals decreases in the subsequent stages of sludge processing, which is a positive phenomenon in terms of its agricultural or natural use.

The above findings confirm that identification of the chemical forms of the heavy metals in municipal sewage sludge is a critical element in ecological risk assessment.

## Figures and Tables

**Table 1:** Operational parameters of the WWTPs.

WWTP	PE *	Average Flow (m^3^·d^−1^)	HRT ** (d)	Wastewater Type	Sludge Dewatering Technique	Hygienizing Agent
WWTP1	143,292	29,140.80	32	domestic and industrial	belt filter press and decanter centrifuge	burned lime
WWTP2	156,667	18,864.17	30	domestic and industrial	decanter centrifuge	burned lime
WWTP3	191,749	19,330.0	21	domestic	filter press	burned lime
WWTP4	11,290	1446.0	20	domestic and industrial	belt filter press	*-*
WWTP5	33,176	4870.0	25	domestic	belt filter press	-

* PE—Population equivalent; ** HRT—Hydraulic retention time of sludge in anaerobic digester. Data were obtained from the concerned WWTPs.

**Table 2:** The Community Bureau of Reference (BCR) sequential extraction procedure [24,25].

Step	Fraction	Extractants	BCR Procedure
F1	Acid soluble/exchangeable fraction; bound to carbonates (mobile)	0.11 M CH_3_COOH (acetic acid)	Add 20 mL CH_3_COOH to 0.5 g of sludge sample. Shake for 16 h.
F2	Reducible fraction; bound to Mn and Fe oxides (mobile)	0.1 M NH_2_OH·HCl (hydroxylamine hydrochloride)	Add 20 mL NH_2_OH·HCl to residue from F1. Shake for 16 h.
F3	Oxidizable fraction; bound to organic matter and sulfides (immobile)	8.8 M H_2_O_2_ (hydrogen peroxide) 1 M CH_3_COONH_4_ (ammonium acetate)	Add 5 mL H_2_O_2_ and incubate at 85 °C for 1 h (repeat twice), and then 25 mL CH_3_COONH_4_ to residue from F2. Shake for 16 h.
F4	Residual fraction (immobile)	HCl/HNO_3_ (3:1) (aqua regia)	Add 5 mL HNO_3_ and 15 mL HCl to residue from F3.

**Table 3:** Heavy metal concentrations in the certified reference material.

Heavy Metal	This Study (mg·kg^−1^)	ERM-CC144 (mg·kg^−1^)	Recovery (%)
Cd	12.0 ± 0.1	14.5	83
Cr	144.1 ± 2.0	168.0	86
Cu	366.9 ± 3.0	348.0	103
Ni	77.1 ± 1.2	91.0	85
Pb	131.5 ± 1.1	157.0	84
Zn	1032.2 ± 3.0	980.0	101
Hg	5.7 ± 0.1	5.9	97

Results are expressed as the mean ± standard deviation.

**Table 4:** Indices for ecological risk assessment.

Index	Equation	Category	Description and Abbreviations	References
Potential Ecological Risk Factor (ER)	$ER=T_{f}^{i}\;\cdot C_{f}$ $T_{f\;}^{i}$—the toxic response factor of metal; C_f_—single metal pollution factor	ER ≤ 40 40 < ER ≤ 80 80 < ER ≤ 160 160 < ER ≤ 320 ER > 320	Low risk (LR) Moderate risk (MR) Considerable risk (CR) High risk (HR) Very high risk (VHR)	[27]
Risk Index (RI)	$RI=\;\sum^{\;}ER$ ER—Potential Ecological Risk Factor	RI ≤ 150 150 < RI ≤ 300 300 < RI ≤ 600 RI > 600	Low risk (LR) Moderate risk (MR) Considerable risk (CR) High risk (HR)	[27]
Risk Assessment Code (RAC)	$RAC=F_{1}$ F1—the percentage share of metal in acid soluble/exchangeable fraction (bound to carbonates)	RAC ≤ 1% 1% < RAC ≤ 10% 10% < RAC ≤ 30% 30% < RAC ≤ 50% RAC > 50%	No risk (NR) Low risk (LR) Medium risk (MR) High risk (HR) Very high risk (VHR)	[28]
Individual Contamination Factor (ICF)	$ICF\;=\frac{F_{1}+F_{2}+F_{3}}{F_{4}}$ F_1_, F_2_, F_3,_ F_4_—the content of metal in all chemical fractions	CF ≤ 1 1 < CF ≤ 3 3 < CF ≤ 6 CF > 6	Low contamination (LC) Moderate contamination (MC) Considerable contamination (CC) Very high contamination (VHC)	[29,30]
Global Risk Index (GRI)	$GRI=\;\sum^{\;}T_{f}^{i}\cdot ICF_{f}$ ICF—Individual Contamination Factor	GRI ≤ 150 150 < GRI ≤ 300 300 < GRI ≤ 600 GRI > 600	Low risk (LR) Moderate risk (MR) Considerable (CR) High risk (HR)	[30]
Individual Ecological Risk (IER)	$IER\;=\frac{F_{1}+F_{2}}{F_{3}+F_{4}}\cdot 100$ F_1_, F_2_, F_3,_ F_4_—the content of metal in all chemical fractions	IER ≤ 50% 50% < IER ≤ 100% 100% < IER ≤ 250% IER > 250%	Low risk (LR) Moderate risk (MR) High risk (HR) Very high risk (VHR)	[This study]
Global Ecological Risk (GER)	$GER\;=\sum^{\;}IER$ IER—Individual Ecological Risk	GER ≤ 100% 100% < GER ≤ 250% 250% < GER ≤ 500% GER > 500%	Low risk (LR) Moderate risk (MR) High risk (HR) Very high risk (VHR)	[This study]

**Table 5:** Physicochemical characteristics of the sewage sludge samples.

Sampling Point	Parameter	Unit	WWTP1	WWTP2	WWTP3	WWTP4	WWTP5
S1	pH	-	6.3	6.7	6.0	6.8	6.4
	Eh	mV	−336.0	−114.0	−333.5	−294.0	−278.0
	DM	g·kg^−1^	31.7	55.2	40.2	33.8	48.5
	OM		24.6	41.2	29.1	24.0	34.7
	Cd	mg·kg^−1^	1.9 ± 0.1	1.4 ± 0.0	3.5 ± 0.1	2.3 ± 0.1	4.6 ± 0.5
	Cr		58.1 ± 4.4	17.8 ± 0.6	46.4 ± 8.0	66.1 ± 0.6	33.6 ± 3.0
	Cu		145.2 ± 6.4	79.7 ± 1.8	139.2 ± 6.3	174.9 ± 3.3	143.5 ± 3.5
	Ni		64.8 ± 1.3	17.0 ± 1.2	35.2 ± 6.1	17.1 ± 0.1	14.6 ± 1.5
	Pb		39.6 ± 0.4	44.1 ± 2.3	132.5 ± 8.4	56.0 ± 1.5	170.9 ± 10.7
	Zn		1013 ± 14.6	774.2 ± 10.3	1603.0 ± 133.6	2248.0 ± 202.7	1379.5 ± 37.6
	Hg		0.4 ± 0.0	0.2 ± 0.0	0.2 ± 0.0	0.3 ± 0.0	0.2 ± 0.1
S2	pH	-	8.9	8.2	8.1	8.4	8.1
	Eh	mV	−349.0	−295.0	−278.0	−304.0	−235.5
	DM	g·kg^−1^	208.4	269.9	177.2	187.4	166.4
	OM		130.0	148.4	106.4	110.8	102.6
	Cd	mg·kg^−1^	4.1 ± 0.2	1.9 ± 0.1	4.7 ± 0.0	4.5 ± 0.2	6.9 ± 0.0
	Cr		70.7 ± 3.2	37.3 ± 1.8	63.6 ± 1.7	120.3 ± 3.9	49.5 ± 0.5
	Cu		252.9 ± 32.2	136.7 ± 5.4	190.6 ± 10.1	280.7 ± 12.1	204.9 ± 2.8
	Ni		126.1 ± 4.0	25.7 ± 0.6	36.3 ± 0.4	29.5 ± 0.6	23.7 ± 0.3
	Pb		59.9 ± 2.9	58.0 ± 1.2	149.7 ± 3.3	121.5 ± 4.6	209.4 ± 6.3
	Zn		1660.0 ± 252.5	1175.9 ± 189.6	2288.2 ± 224.8	3448.5 ± 91.7	2184.5 ± 68.4
	Hg		0.4 ± 0.0	0.3 ± 0.1	0.2 ± 0.0	1.2 ± 0.0	0.2 ± 0.0

Results are expressed as the mean ± standard deviation.

**Table 6:** Heavy metal concentrations in individual fractions of the sewage sludge samples.

**S1**	**Fraction**	**Cd**	**Cr**	**Cu**	**Ni**	**Pb**	**Zn**	**Hg**
mg·kg^−1^_DM_								
WWTP1	F1	0.3 ± 0.0	1.4 ± 0.1	9.4 ± 0.3	25.6 ± 1.0	BLOQ	306.3 ± 10.2	BLOQ
	F2	0.8 ± 0.0	1.9 ± 0.2	2.2 ± 0.7	9.0 ± 1.4	BLOQ	246.2 ± 29.3	BLOQ
	F3	0.7 ± 0.1	51.9 ± 9.6	136.7 ± 19.5	24.8 ± 3.6	41.9 ± 5.3	160.4 ± 21.4	BLOQ
	F4	BLOQ	10.9 ± 1.9	BLOQ	3.5 ± 0.6	4.2 ± 1.0	7.3 ± 1.2	0.1 ± 0.0
	R; %	95.0	113.4	101.9	97.2	116.5	71.1	34.8
WWTP2	F1	0.2 ± 0.0	1.0 ± 0.1	4.5 ± 0.4	4.2 ± 0.7	0.1 ± 0.2	192.7 ± 17.9	BLOQ
	F2	0.7 ± 0.1	BLOQ	BLOQ	2.4 ± 0.2	0.2 ± 0.5	271.6 ± 9.0	BLOQ
	F3	0.7 ± 0.1	12.7 ± 0.7	97.3 ± 2.6	8.2 ± 0.4	49.6 ± 2.4	169.8 ± 12.3	BLOQ
	F4	0.03 ± 0.0	7.4 ± 1.2	BLOQ	2.7 ± 0.2	7.1 ± 0.8	23.9 ± 1.4	0.1 ± 0.0
	R; %	107.6	118.3	127.7	103.1	129.5	85.0	56.7
WWTP3	F1	0.1 ± 0.1	1.0 ± 0.1	5.4 ± 0.4	17.2 ± 2.3	BLOQ	443.2 ± 26.5	BLOQ
	F2	2.1 ± 0.3	0.5 ± 0.5	0.9 ± 1.0	7.6 ± 3.1	BLOQ	594.9 ± 172.2	BLOQ
	F3	1.7 ± 0.3	42.7 ± 6.4	152.5 ± 14.9	13.2 ± 1.3	77.9 ± 25.5	408.7 ± 21.4	BLOQ
	F4	BLOQ	11.8 ± 1.9	BLOQ	3.8 ± 0.3	52.0 ± 13.5	52.4 ± 6.1	0.1 ± 0.0
	R; %	110.0	120.4	113.9	118.5	98.7	93.2	50.4
WWTP4	F1	BLOQ	1.6 ± 0.1	3.3 ± 0.1	4.1 ± 0.1	BLOQ	538.5 ± 38.4	BLOQ
	F2	1.4 ± 0.1	1.2 ± 0.1	BLOQ	2.3 ± 0.1	BLOQ	998.1 ± 85.8	BLOQ
	F3	1.1 ± 0.1	65.7 ± 1.3	216.5 ± 5.4	6.5 ± 0.4	14.3 ± 3.3	499.1 ± 25.3	BLOQ
	F4	BLOQ	16.1 ± 1.1	BLOQ	3.8 ± 0.1	35.2 ± 4.6	16.7 ± 2.9	0.1 ± 0.0
	R; %	109.2	128.0	125.7	97.6	88.4	91.8	36.5
WWTP5	F1	0.9 ± 0.2	1.0 ± 0.1	8.6 ± 2.2	5.2 ± 0.1	BLOQ	477.9 ± 15.1	BLOQ
	F2	3.3 ± 0.1	BLOQ	4.0 ± 0.3	2.6 ± 0.5	BLOQ	540.7 ± 19.7	BLOQ
	F3	1.4 ± 0.1	23.9 ± 1.2	129.1 ± 6.4	4.9 ± 0.3	38.1 ± 6.3	228.1 ± 19.5	BLOQ
	F4	BLOQ	13.4 ± 0.4	7.8 ± 0.7	3.8 ± 0.2	124.9 ± 6.3	85.8 ± 4.5	0.1 ± 0.0
	R; %	122.8	114.4	104.2	113.1	95.5	96.6	69.7
S2	**Fraction**	**Cd**	**Cr**	**Cu**	**Ni**	**Pb**	**Zn**	**Hg**
WWTP1	F1	0.3 ± 0.3	1.3 ± 0.2	4.4 ± 0.2	41.6 ± 2.4	BLOQ	292.8 ± 49.6	BLOQ
	F2	1.8 ± 0.2	0.4 ± 0.4	BLOQ	14.2 ± 1.6	BLOQ	496.1 ± 44.1	BLOQ
	F3	3.0 ± 0.2	61.6 ± 4.6	264.8 ± 31.6	73.1 ± 6.1	35.4 ± 3.0	549.4 ± 33.3	BLOQ
	F4	BLOQ	22.8 ± 2.1	9.6 ± 2.1	7.7 ± 0.8	35.9 ± 3.1	32.6 ± 2.2	0.5 ± 0.1
	R; %	123.9	121.6	110.2	108.3	119.2	83.1	113.1
WWTP2	F1	BLOQ	0.8 ± 0.0	2.5 ± 0.3	5.8 ± 0.2	BLOQ	268.6 ± 11.3	BLOQ
	F2	1.1 ± 0.0	BLOQ	BLOQ	3.3 ± 0.2	BLOQ	378.8 ± 10.0	BLOQ
	F3	1.4 ± 0.1	29.2 ± 0.8	165.1 ± 9.6	12.9 ± 1.4	53.7 ± 3.0	306.8 ± 15.5	BLOQ
	F4	BLOQ	18.7 ± 0.9	9.2 ± 1.1	6.6 ± 0.4	31.0 ± 2.2	62.7 ± 2.8	0.3 ± 0.0
	R; %	131.2	130.9	129.5	111.2	146.1	87.6	100.6
WWTP3	F1	BLOQ	1.0 ± 0.1	6.0 ± 0.3	13.0 ± 0.2	BLOQ	630.1 ± 13.0	BLOQ
	F2	2.6 ± 0.1	BLOQ	BLOQ	5.4 ± 0.2	BLOQ	823.3 ± 32.8	BLOQ
	F3	3.3 ± 0.1	59.6 ± 1.0	229.0 ± 0.6	15.1 ± 0.4	48.9 ± 0.7	875.9 ± 36.0	BLOQ
	F4	BLOQ	27.0 ± 0.7	12.0 ± 0.7	7.0 ± 0.1	130.3 ± 3.4	101.9 ± 5.7	0.2 ± 0.2
	R; %	125.4	137.8	129.8	111.5	119.8	106.7	90.7
WWTP4	F1	1.0 ± 0.0	1.1 ± 0.1	8.8 ± 1.2	8.2 ± 0.2	BLOQ	1258.7 ± 58.2	BLOQ
	F2	1.8 ± 0.0	BLOQ	BLOQ	1.5 ± 0.1	BLOQ	880.5 ± 37.4	BLOQ
	F3	3.5 ± 0.2	105.2 ± 5.0	304.5 ± 12.8	13.9 ± 0.4	46.8 ± 2.2	1502.8 ± 31.1	BLOQ
	F4	BLOQ	55.6 ± 1.5	24.4 ± 2.6	9.3 ± 0.3	102.0 ± 3.2	70.9 ± 4.9	0.4 ± 0.1
	R; %	138.4	134.5	120.3	111.2	122.5	107.7	31.2
WWTP5	F1	1.3 ± 0.1	0.9 ± 0.0	7.6 ± 0.7	8.5 ± 0.1	BLOQ	629.9 ± 15.5	BLOQ
	F2	3.9 ± 0.4	BLOQ	BLOQ	2.4 ± 0.1	BLOQ	725.8 ± 14.0	BLOQ
	F3	3.9 ± 0.1	37.0 ± 1.1	208.6 ± 4.9	8.1 ± 1.8	33.8 ± 3.5	735.3 ± 26.0	BLOQ
	F4	BLOQ	24.3 ± 2.0	10.4 ± 0.7	6.6 ± 0.5	188.3 ± 7.9	111.7 ± 4.7	0.2 ± 0.1
	R; %	132.5	125.7	110.6	108.3	106.1	100.9	96.7

Results are expressed as the mean ± standard deviation; R—heavy metal recovery rate (%); BLOQ—below limit of quantification.

**Table 7:** Ecological risks of the heavy metals in the thickened sewage sludge samples.

WWTP	Index	Cd	Cr	Cu	Ni	Pb	Zn	Hg
WWTP1	ER	**582.0** (**VHR**)	1.2 (LR)	13.2 (LR)	16.2 (LR)	13.2 (LR)	14.5 (LR)	**231.5** (**HR**)
	RI	**871.7** (**HR**)						
	RAC; %	**17.7** (**MR**)	2.0 (LR)	6.3 (LR)	**40.6** (**HR**)	0.0 (NR)	**42.5** (**HR**)	0.0 (NR)
	ICF	-	-	-	**16.8** (**VHC**)	-	**97.6** (**VHC**)	-
	GRI	**181.5** (**MR**)						
	IER; %	**173.0** (**HR**)	5.2 (LR)	8.5 (LR)	**121.9** (**HR**)	0.0 (LR)	**329.4** (**VHR**)	0.0 (LR)
	GER; %	**637.9** (**VHR**)						
WWTP2	ER	**435.0** (**VHR**)	0.4 (LR)	7.2 (LR)	4.3 (LR)	14.7 (LR)	11.1 (LR)	**103.7** (**CR**)
	RI	576.4 (CR)						
	RAC; %	**10.7** (**MR**)	4.6 (LR)	4.4 (LR)	**24.1** (**MR**)	0.2 (NR)	**29.3** (**MR**)	0.0 (NR)
	ICF	-	-	-	**5.5** (**CC**)	-	**26.6** (**VHC**)	-
	GRI	54.1 (LR)						
	IER; %	**127.0** (**HR**)	4.8 (LR)	4.6 (LR)	**61.4** (**MR**)	0.5 (LR)	**239.7** (**VHR**)	0.0 (LR)
	GER; %	**438.1** (**HR**)						
WWTP3	ER	**1060.4** (**VHR**)	0.9 (LR)	12.7 (LR)	8.8 (LR)	**44.2 (MR)**	22.9 (LR)	**103.0** (**CR**)
	RI	**1252.9** (**HR**)						
	RAC; %	2.4 (LR)	1.7 (LR)	3.4 (LR)	**41.4** (**HR**)	0.0 (NR)	**29.6** (**MR**)	0.0 (NR)
	ICF	-	-	-	**9.9** (**VHC**)	-	**27.6** (**VHC**)	-
	GRI	77.3 (LR)						
	IER; %	**133.2** (**HR**)	2.6 (LR)	4.1 (LR)	**145.2** (**HR**)	0.0 (LR)	**225.2** (**HR**)	0.0 (LR)
	GER; %	**510.4** (**VHR**)						
WWTP4	ER	**692.4** (**VHR**)	1.3 (LR)	15.9 (LR)	4.3 (LR)	18.7 (LR)	32.1 (LR)	**154.1**(**CR**)
	RI	**918.7** (**HR**)						
	RAC; %	0.0 (NR)	1.9 (LR)	1.5 (LR)	**24.4** (**MR**)	0.0 (NR)	**26.2** (**MR**)	0.0 (NR)
	ICF	-	-	-	**3.4** (**CC**)	-	**121.7** (**VHC**)	-
	GRI	138.4 (LR)						
	IER; %	**133.2** (**HR**)	3.4 (LR)	1.5 (LR)	**61.6** (**MR**)	0.0 (LR)	**297.9** (**VHR**)	0.0 (LR)
	GER; %	**497.6** (**HR**)						
WWTP5	ER	**1384.8** (**VHR**)	0.7 (LR)	13.0 (LR)	3.6 (LR)	**57.0 (MR)**	19.7 (LR)	**103.0** (**CR**)
	RI	**1581.8** (**HR**)						
	RAC; %	**16.4** (**MR**)	2.6 (LR)	5.8 (LR)	**31.4** (**HR**)	0.0 (NR)	**35.9** (**HR**)	0.0 (NR)
	ICF	-	-	-	**3.3 (CC)**	-	**14.5** (**VHC**)	-
	GRI	31.2 (LR)						
	IER; %	**298.4** (**VHR**)	2.7 (LR)	9.2 (LR)	**88.8** (**MR**)	0.0 (LR)	**324.6** (**VHR**)	0.0 (LR)
	GER; %	**723.7** (**VHR**)						

Bold indicates the highest levels.

**Table 8:** Ecological risks of the heavy metals in the dewatered and/or hygienized sewage sludge samples.

WWTP	Index	Cd	Cr	Cu	Ni	Pb	Zn	Hg
WWTP1	ER	**1231.0** (**VHR**)	1.4 (LR)	23.0 (LR)	31.5 (LR)	20.0 (LR)	23.7 (LR)	**238.3** (**HR**)
	RI	**1568.9** (**HR**)						
	RAC; %	6.2 (LR)	1.5 (LR)	1.6 (LR)	**30.4** (**HR**)	0.0 (NR)	**21.4 (MR)**	0.0 (NR)
	ICF	-	-	-	**16.7** (**VHC**)	-	**41.1** (**VHC**)	-
	GRI	**124.8** (**MR**)						
	IER; %	**68.9** (**MR**)	1.9 (LR)	1.6 (LR)	**69.1** (**MR**)	0.0 (NR)	**135.6** (**HR**)	0.0 (LR)
	GER; %	**277.1** (**HR**)						
WWTP2	ER	**557.8** (**VHR**)	0.7 (LR)	12.4 (LR)	6.4 (LR)	19.3 (LR)	16.8 (LR)	**149.6** (**CR**)
	RI	**763.1** (**HR**)						
	RAC; %	0.0 (NR)	1.7 (LR)	1.4 (LR)	**20.3 (MR)**	0.0 (NR)	**26.4 (MR)**	0.0 (NR)
	ICF	-	-	-	**3.3** (**CC**)	-	**15.2** (**VHC**)	-
	GRI	31.9 (LR)						
	IER; %	**80.1** (**MR**)	1.7 (LR)	1.4 (LR)	46.7 (LR)	0.0 (LR)	**175.2** (**HR**)	0.0 (LR)
	GER; %	**305.2** (**HR**)						
WWTP3	ER	**1402.2** (**VHR**)	1.3 (LR)	17.3 (LR)	9.1 (LR)	**49.9** (**MR**)	32.7 (LR)	**138.6** (**CR**)
	RI	**1651.1** (**HR**)						
	RAC; %	0.0 (NR)	1.1 (LR)	2.4 (LR)	**32.0** (**HR**)	0.0 (NR)	**25.9 (MR)**	0.0 (NR)
	ICF	-	-	-	**4.8** (**CC**)	-	**22.9** (**VHC**)	-
	GRI	46.7 (LR)						
	IER; %	**77.8** (**MR**)	1.2 (LR)	2.5 (LR)	**83.1** (**MR**)	0.0 (LR)	**148.6** (**HR**)	0.0 (LR)
	GER; %	**313.1** (**HR**)						
WWTP4	ER	**1356.7** (**VHR**)	2.4 (LR)	25.5 (LR)	7.4 (LR)	**40.5** (**MR**)	**49.3 (MR)**	**691.1** (**VHR**)
	RI	**2172.8** (**HR**)						
	RAC; %	**15.6** (**MR**)	0.7 (NR)	2.6 (LR)	**24.9 (MR)**	0.0 (NR)	**33.9** (**HR**)	0.0 (NR)
	ICF	-	-	-	**2.5** (**MC**)	-	**51.4** (**VHC**)	-
	GRI	64.0 (LR)						
	IER; %	**80.8** (**MR**)	0.7 (LR)	2.7 (LR)	41.7 (LR)	0.0 (LR)	**135.9** (**HR**)	0.0 (LR)
	GER; %	**261.8** (**HR**)						
WWTP5	ER	**2082.9** (**VHR**)	1.0 (LR)	18.6 (LR)	5.9 (LR)	**69.8** (**MR**)	31.2 (LR)	**124.4** (**CR**)
	RI	**2333.8** (**HR**)						
	RAC; %	**14.3** (**MR**)	1.5 (LR)	3.3 (LR)	**33.2** (**HR**)	0.0 (NR)	**28.6** (**MR**)	0.0 (NR)
	ICF	-	-	-	**2.9** (**MC**)	-	**18.7** (**VHC**)	-
	GRI	33.0 (LR)						
	IER; %	**133.2** (**HR**)	1.5 (LR)	3.5 (LR)	**74.5** (**MR**)	0.0 (LR)	**160.1** (**HR**)	0.0 (LR)
	GER; %	**372.7** (**HR**)						

Bold indicates the highest levels.

**Table 9:** Pearson’s correlation matrix for the analyzed variables.

**S1**	**Cd**	**Cr**	**Cu**	**Ni**	**Pb**	**Zn**	**Hg**	**pH**	**Eh**	**DM**	**OM**
Cd	1.00										
Cr	−0.04	1.00									
Cu	0.36	0.89 *	1.00								
Ni	−0.26	0.44	0.16	1.00							
Pb	0.98 *	−0.20	0.19	−0.34	1.00						
Zn	0.29	0.68 *	0.82 *	−0.29	0.20	1.00					
Hg	−0.39	0.65*	0.39	0.78 *	−0.55	−0.03	1.00				
pH	−0.50	−0.14	−0.20	−0.46	−0.50	0.01	0.01	1.00			
Eh	−0.42	−0.78 *	−0.83 *	−0.54	−0.29	−0.50	−0.42	0.65 *	1.00		
DM	0.10	−0.94 *	−0.79 *	−0.61	0.26	−0.49	−0.74 *	0.30	0.80 *	1.00	
OM	0.00	−0.95 *	−0.86 *	−0.51	0.17	−0.60	−0.66 *	0.30	0.83 *	0.99 *	1.00
**S2**	Cd	Cr	Cu	Ni	Pb	Zn	Hg	pH	Eh	DM	OM
Cd	1.00										
Cr	0.16	1.00									
Cu	0.40	0.86 *	1.00								
Ni	−0.10	0.08	0.41	1.00							
Pb	0.89 *	0.01	0.08	−0.52	1.00						
Zn	0.47	0.87 *	0.73 *	−0.28	0.45	1.00					
Hg	−0.02	0.94 *	0.72 *	−0.06	−0.11	0.78 *	1.00				
pH	−0.16	0.15	0.18	0.59	−0.42	−0.19	0.16	1.00			
Eh	0.54	−0.34	−0.38	−0.76 *	0.82 *	0.14	−0.33	−0.50	1.00		
DM	−0.84 *	−0.36	−0.52	0.05	−0.75 *	−0.64 *	−0.12	0.13	−0.45	1.00	
OM	−0.80 *	−0.34	−0.41	0.24	−0.80 *	−0.68 *	−0.13	0.21	−0.57	0.98 *	1.00

* Significant correlation at *p* < 0.05.

**Table 10:** Pearson’s correlation matrices of the ecological risk levels.

S1				S2			
	ER_Cd	RAC_Cd	IER_Cd		ER_Cd	RAC_Cd	IER_Cd
ER_Cd	1.00			ER_Cd	1.00		
RAC_Cd	0.07	1.00		RAC_Cd	0.64 *	1.00	
IER_Cd	0.74 *	0.56	1.00	IER_Cd	0.69 *	0.98 *	1.00
	ER_Cr	RAC_Cr	IER_Cr		ER_Cr	RAC_Cr	IER_Cr
ER_Cr	1.00			ER_Cr	1.00		
RAC_Cr	0.03	1.00		RAC_Cr	−0.88 *	1.00	
IER_Cr	−0.32	0.09	1.00	IER_Cr	−0.60	0.88 *	1.00
	ER_Cu	RAC_Cu	IER_Cu		ER_Cu	RAC_Cu	IER_Cu
ER_Cu	1.00			ER_Cu	1.00		
RAC_Cu	−0.33	1.00		RAC_Cu	0.22	1.00	
IER_Cu	−0.39	−0.32	1.00	IER_Cu	0.22	0.99 *	1.00
	ER_Ni	RAC_Ni	IER_Ni		ER_Ni	RAC_Ni	IER_Ni
ER_Ni	1.00			ER_Ni	1.00		
RAC_Ni	0.76 *	1.00		RAC_Ni	0.21	1.00	
IER_Ni	0.69 *	0.93 *	1.00	IER_Ni	0.22	0.89 *	1.00
	ER_Zn	RAC_Zn	IER_Zn		ER_Zn	RAC_Zn	IER_Zn
ER_Zn	1.00			ER_Zn	1.00		
RAC_Zn	−0.49	1.00		RAC_Zn	0.78 *	1.00	
IER_Zn	0.05	0.59	1.00	IER_Zn	−0.46	0.09	1.00

* Significant correlation at *p* < 0.05.

**Table 11:** Loadings of the variables on the significant principal components for the heavy metals in the thickened sewage sludge samples.

Variables	PC1	PC2	PC3
Cd	0.17	**−0.97**	−0.13
Cr	**0.88**	0.12	0.45
Cu	**0.93**	−0.24	0.24
Ni	−0.03	0.11	**0.96**
Pb	0.02	**−0.97**	−0.24
Zn	**0.94**	−0.10	−0.29
Hg	0.29	0.36	**0.84**
Eigen value	3.01	2.73	1.06
Variability (%)	43.06	39.00	15.11
Cumulative (%)	43.06	82.05	97.16

Bold values indicate a significant correlation.

**Table 12:** Loadings of the variables on the significant principal components for the heavy metals in the dewatered and/or hygienized sewage sludge samples.

Variables	PC1	PC2	PC3
Cd	0.13	**0.98**	0.08
Cr	**0.99**	0.03	0.09
Cu	**0.83**	0.27	0.48
Ni	−0.01	−0.18	**0.98**
Pb	0.01	**0.93**	−0.36
Zn	**0.88**	0.39	−0.21
Hg	**0.97**	−0.16	−0.08
Eigen value	3.62	2.14	1.14
Variability (%)	51.74	30.64	16.26
Cumulative (%)	51.74	82.38	98.64

Bold values indicate significant correlation.
